# Morphological evidence for a neurotensinergic periaqueductal gray-rostral ventromedial medulla-spinal dorsal horn descending pathway in rat

**DOI:** 10.3389/fnana.2014.00112

**Published:** 2014-10-09

**Authors:** Jian Wang, Hua Zhang, Yu-Peng Feng, Hua Meng, Li-Ping Wu, Wen Wang, Hui Li, Ting Zhang, Jin-Shan Zhang, Yun-Qing Li

**Affiliations:** ^1^Department of Anatomy, Histology and Embryology & K.K. Leung Brain Research Centre, Preclinical School of Medicine, The Fourth Military Medical UniversityXi’an, China; ^2^Department of Geriatrics, Xijing Hospital, The Fourth Military Medical UniversityXi’an, China

**Keywords:** neurotensin, NTR2, periaqueductal gray matter, rostral ventromedial medulla (RVM), nociception, analgesia

## Abstract

Neurotensin (NT) is an endogenous neuropeptide that exerts potent opioid-independent analgesic effects, most likely via the type 2 NT receptor (NTR2). Previous morphological and electrophysiological studies suggested that the NT-NTR2 system is primarily localized in structures that constitute the descending pain control pathway, such as the periaqueductal gray (PAG), the rostral ventromedial medulla (RVM), and the spinal dorsal horn (SDH). However, relevant morphological evidence for this neurotensinergic (NTergic) circuit is lacking. Thus, the aim of the present study was to morphologically elucidate the potential sites and connections in the NT-NTR2 system that are involved in the descending pain control pathway. Based on light and electron microscopy combined with anterograde and retrograde tracing, we found evidence that NTR2-immunoreactive (IR) neurons in the RVM receive NT-IR projections originating from the PAG; express NT, serotonin (5-HT), or both; and send projections that terminate in laminae I and II of the SDH. These results suggest that NTR2 may contribute to pain control by binding to NT in the PAG-RVM-SDH pathway. In conclusion, our data provide morphological evidence for an NTergic PAG-RVM-SDH pathway, implicating novel mechanisms of NT-induced analgesia.

## Introduction

Pain is most likely one of the most prevalent human health problems, contributing to individual morbidity and mortality and imposing high societal costs (Mogil, 2013). However, due to their limited efficacy and large numbers of side effects, the current analgesics for pain remain far from optimal. The most common reason for these limitations is that the mechanisms underlying the modulation of nociception in the central nervous system (CNS) remain poorly understood. Elucidating the nerve fiber connections and their chemical properties will be beneficial for determining these pain modulation mechanisms.

The periaqueductal gray (PAG) modulates nociception via a descending pathway that relays in the rostral ventromedial medulla (RVM) and terminates in superficial laminae (laminae I and II) of the spinal dorsal horn (SDH; Morgan et al., 2008), referred to as the PAG-RVM-SDH pathway. This pathway comprises an essential neural circuit that exerts powerful modulatory influences on pain (Basbaum and Fields, 1984). Based on morphological, behavioral, and electrophysiological evidence, various neurotransmitters, such as GABA and serotonin (5-HT), are involved in this pathway, thereby contributing to analgesia under both physiological and pathological conditions (Yang et al., 2002; Taylor and Basbaum, 2003; Buhler et al., 2005; Morgan et al., 2008; Mitchell et al., 2009). However, the roles of other neurotransmitters, including neurotensin (NT), are not fully supported by these experimental data.

NT is a tridecapeptide that was originally isolated from the bovine hypothalamus in 1973 (Carraway and Leeman, 1973). In the CNS, NT exerts many potent effects, including analgesia, hypothermia, dopaminergic neurotransmission regulation, anterior pituitary hormone secretion stimulation, and cancer cell growth promotion (White et al., 2012). NT and its analogs exerted dose-dependent analgesic effects in both somatic and visceral pain paradigms (Dobner, 2006). To date, three subtypes of NT receptors (NTRs) have been identified. Both NTR1 and NTR2 are G-protein-coupled receptors that mediate NT-induced signal transduction (Vincent et al., 1999; Mazella and Vincent, 2006). NTR3 is a single transmembrane receptor that shares 100% homology with the sorting protein gp95/sortilin, and its function is unknown (Mazella, 2001).

Previous neuropharmacology studies have demonstrated that the analgesic effects of NT are primarily mediated by NTR1 and NTR2 (Dubuc et al., 1994; Gully et al., 1997; Pettibone et al., 2002; Remaury et al., 2002; Dobner, 2006; Buhler et al., 2008). It has been previously reported that NTR1 expressed by spinally projecting serotonergic neurons in the RVM partially contributed to the descending inhibitory modulation of NT-induced antinociception (Buhler et al., 2005). However, other lines of evidence suggest that NTR2 is also implicated in NT-mediated analgesia, but its role has not yet been directly or morphologically elucidated. The analgesic effects produced by intracerebroventricular administration of NT are not inhibited by the NTR1-specific antagonist SR48692 but are blocked by SR142948A, which recognizes both NTR1 and NTR2 (Gully et al., 1993, 1997; Dubuc et al., 1994; Buhler et al., 2008). Additionally, receptor knock-down strategies utilizing antisense oligonucleotides also suggest a role of NTR2 in antinociception (Dubuc et al., 1999; Maeno et al., 2004). However, it remains unclear how these NT-NTR2 interactions contribute to NT-mediated analgesia. Moreover, our previous study and other studies suggest that 5-HT, which is strongly expressed in descending projections from the RVM, acts as a neurotransmitter that regulates nociceptive transmission in the superficial laminae of the SDH (Li et al., 2002a; Buhler et al., 2005). Therefore, based on these previous findings, we hypothesized that NT may participate in antinociception via a pathway in which NT released from NT-containing terminals of neurons originating from the PAG regulates the activities of NTergic and/or serotonergic spinally projecting neurons by binding to NTR2 on these RVM neurons, modulating neurotransmitter release in the SDH.

Accordingly, to examine the detailed morphological bases of our hypothesis regarding the RVM-PAG-SDH pathway, we performed the current study to search for morphological evidence of this pathway via both light and electron microscopy.

## Methods

### Animals

A total of 36 adult male Sprague-Dawley (SD) rats (weighing 250–300 g) were used in the present study. The Ethics Committee for Animal Experiments of the Fourth Military Medical University (Xi’an, P. R. China) approved all animal experiments (Permit number: 10071). Efforts were made to minimize the number and suffering of the animals used.

### Groups

In the present study, rats were randomly separated into three groups for different purposes. In group 1, the anterograde tracer biotinylated dextran amine (BDA) was injected into the PAG and the retrograde tracer fluoro-gold (FG) was injected into the SDH, followed by immunofluorescence histochemical staining to reveal the distributions of the RVM-projecting fibers originating from the PAG, the SDH-projecting neurons located in the RVM, and the connections between the PAG-originating NTergic fibers and the SDH-projecting NTR2-immunoreactive (IR) neurons in the RVM. In group 2, the anterograde tracer BDA was injected into the PAG and the retrograde tracer horseradish peroxidase (HRP) was injected into the SDH, followed by electron microscopy analysis to further determine whether the connections between the neurons noted form actual synapses. In group 3, after injection of the retrograde tracer FG into the SDH, colchicine was administered to the animals, followed by immunofluorescence histochemical staining to identify the neurochemical properties of the SDH-projecting neurons in the RVM.

### Anterograde and retrograde tracer injection

*BDA injection*. In the first two groups, the anterograde tracer BDA was used to identify the RVM-projecting fibers and terminals originating from the PAG. The procedures for BDA injection were essentially the same as those previously described by our group (Niu et al., 2009). Briefly, 0.2 µl of 10% BDA (molecular weight 10,000 Da; Molecular Probes, Eugene, OR, USA) dissolved in 0.9% saline was stereotaxically pressure-injected into the right lateral PAG (LPAG) (8.0 mm posterior to bregma, 0.85 mm lateral to the midline, and 5.4 mm deep into the cerebral surface) according to the rat brain atlas (Paxinos and Watson, 2005).

*FG/HRP injection*. For light microscopy analysis of groups 1 and 3, FG was used as a retrograde tracer to label the neurons that send projections to the SDH, whereas HRP served as an appropriate retrograde tracer for electron microscopy analysis of group 2. The procedures for FG/HRP injection were identical to those used in our previous studies (Yang et al., 2002; Lü et al., 2009; Dong et al., 2011). Briefly, for light microscopy analysis of group 1, after exposing the cervical cord, 0.1 µl of 4% FG (Fluorochrome, Denver, CO, USA) dissolved in 0.9% saline was stereotaxically pressure-injected into the right side of the cervical dorsal horn immediately after BDA injection. In group 3, a similar procedure for a single FG injection into the SDH was performed as in group 1. For electron microscopy analysis in group 2, 3 days after BDA injection, the rats were re-anesthetized and injected with 0.1–0.3 µl of 30% HRP (Toyobo, Osaka, Japan) dissolved in 0.9% saline into the right side of the cervical dorsal horn.

An adequate survival period was established to allow for the transportation of the tracers. The rats in groups 1 and 3 were allowed to recover for 6 days before perfusion, whereas the rats in group 2 were allowed to recover for 3 days after the injection of HRP.

### Colchicine treatment

The rats in group 3 that had previously received a single FG injection into the SDH were placed in the stereotaxic instrument again, and 5 µl of colchicine (10 µg/µl; Sigma, St. Louis, MO, USA) was injected into the lateral ventricle 2 days before perfusion. The purpose of intracerebroventricular administration of colchicine was to effectively block axoplasmic transport of the CNS neurons, thereby markedly increasing the somatic retention of neuropeptides, such as NT (Tsukahara and Yamanouchi, 2003). Therefore, the NT-IR neurons in the RVM, rather than the corresponding fibers and terminals, were examined.

### Immunohistochemical and immunofluorescence labeling and analyses

The rats in groups 1 and 3 were transcardially perfused with 150 ml of 0.9% saline, followed by 500 ml of 4% paraformaldehyde in 0.1 M phosphate buffer (PB, pH 7.4). The brains and/or spinal cords were transversely sliced into 30-µm-thick coronal sections using a freezing microtome (CM1950, Leica, Heidelberg, Germany), and the sections were serially collected into six dishes containing 0.01 M phosphate-buffered saline (PBS, pH 7.4) as six sets of every sixth serial section.

The sections in the first dish for both groups (groups 1 and 3) were used to evaluate the FG injection sites in the SDH and the distribution patterns of the retrogradely FG-labeled neurons in the RVM under an epifluorescence microscope (BX-60; Olympus, Tokyo, Japan) using an appropriate filter for FG (excitation 350–395 nm; emission 430 nm). Then, these sections were processed for Nissl staining.

To examine the BDA injection site and the distribution of anterogradely BDA-labeled fibers and terminals, the sections in the second dish of group 1 were treated with an Avidin/Biotin Kit (1:200, SP-2001; Vector Labs, Burlingame, CA, USA). Then, BDA was visualized using the chromogen diaminobenzidine (DAB). Finally, the sections were observed under a light microscope (AH-3; Olympus, Tokyo, Japan).

The sections in the third, fourth, and fifth dishes of group 1 were used to evaluate the triple-labeling of FG/NTR2/BDA, FG/NT/BDA, and NTR2/NT/BDA, respectively. All of the antisera used in this study are presented in Table 1. The sections were incubated at room temperature in primary antisera in 0.01 M PBS containing 5% normal donkey serum (NDS), 0.3% Triton X-100, 0.05% NaN_3_, and 0.25% carrageenan (PBS-NDS, pH 7.4) for 24 h. Then, the sections were incubated in fluorescein-labeled IgG (second antisera) for 6 h.

**Table 1:** **Antisera used in each group**.

Methods	Purpose	Primary antiserum	Secondary antiserum	Tertiary antiserum
Light microscopy	NTR2/BDA/FG	Rabbit anti-NTR2 (1:1000, Millipore, Temecula, CA, USA) Guinea pig anti-FG (1:100, Protos Biotech, New York, NY, USA)	Alexa594 donkey anti-rabbit (1:500, Invitrogen, Camarillo, CA, USA) Alexa488 goat anti- guinea pig (1:500, Invitrogen) Alexa647 avidin (1:1000, Invitrogen)
	FG/BDA/NT	Guinea pig anti-FG (1:100, Protos Biotech) Rat anti-NT (1:200, Protos Biotech)	Alexa488 goat anti-guinea pig (1:500, Invitrogen) Alexa594 goat anti-rat (1:500, Invitrogen) Alexa647 avidin (1:1000, Invitrogen)
	NTR2/BDA/NT	Rabbit anti-NTR2 (1:1000, Millipore) Rat anti-NT (1:200, Protos Biotech)	Alexa488 donkey anti-rabbit (1:500, Invitrogen) Alexa594 goat anti- rat (1:500, Invitrogen) Alexa647 avidin (1:1000, Invitrogen)
	NTR2/NT/FG	Rabbit anti-NTR2 (1:1000, Millipore) Rat anti-NT (1:200, Protos Biotech) Guinea pig anti-FG (1:100, Protos Biotech)	Biotinylated donkey anti-rabbit (1:500, Millipore) Alexa594 goat anti-rat (1:500, Invitrogen) Alexa488 goat anti-guinea pig (1:500, Invitrogen)	Alexa647 avidin (1:1000, Invitrogen)
	NTR2/NT/5-HT	Rabbit anti-NTR2 (1:1000, Millipore) Rat anti-NT (1:200, Protos Biotech) Goat anti-5-HT (1:500, Immunostar, Hudson, WI, USA)	Biotinylated donkey anti-rabbit (1:500, Millipore) Cy3 donkey anti-rat (1:500, Millipore) Alexa647 donkey anti-goat (1:500, Invitrogen)	FITC-avidin (1:1000, Vector Labs)
Electron microscopy	NT/NTR2/HRP*	Rabbit anti-NTR2 (1:1000, Millipore) Rat anti-NT (1:200, Protos Biotech)	Biotinylated donkey anti-Rat IgG (1:200, Millipore) Goat anti-rabbit IgG conjugated to 1.4-nm gold particles (1:100; Nanoprobes)	Avidin-biotinylated peroxidase complex (1:50, Vector Labs)
	NTR2/BDA/HRP*	Rabbit anti-NTR2 (1:1000, Millipore)	Goat anti-rabbit IgG conjugated to 1.4-nm gold particles (1:100; Nanoprobes)	Avidin-biotinylated peroxidase complex (1:50, Vector Labs)
	NT/BDA/HRP*	Rat anti-NT (1:200, Protos Biotech)	Goat anti-rat IgG conjugated to 1.4-nm gold particles (1:100; Nanoprobes)	Avidin-biotinylated peroxidase complex (1:50, Vector Labs)

*The DAB reaction utilized for the demonstration of HRP in each group was performed before the immuno-electron microscopy procedures.

The sections in the second and third dishes of group 3 were processed for triple-labeling of FG/NT/NTR2 and NTR2/NT/5-HT, respectively. Different antisera (Table 1) were used for triple-immunofluorescence histochemical staining. Similarly, the sections were sequentially incubated at room temperature in primary antisera for 24 h and the secondary antisera for 6 h, followed by incubation in fluorophore-conjugated avidin D for 2 h.

The sections in the sixth dish were used for control experiments. The procedures were the same as those described above except that the primary antisera were omitted or were replaced with normal serum from the same species. No immunopositive products were detected.

After immunofluorescence histochemical staining, the sections were observed and images were captured under a confocal laser-scanning microscope (CLSM, FV1000, Olympus, Tokyo, Japan). Digital images were captured using FLUOVIEW software (Olympus).

For immunofluorescence histochemical staining analysis of NTR2/NT/FG in the RVM, the neurons were counted to estimate the co-localization of the markers. Seven transverse sections selected from each animal were analyzed to provide an adequate sample size and to ensure that the entire RVM was examined. The sections were selected at the following distances caudal to bregma (Paxinos and Watson, 2005): 11.4, 11.16, 10.92, 10.68, 10.44, 10.2, and 9.96 mm. Neuronal counting rather than stereological methods were used, providing potentially biased estimates of the total number of neurons in each group. Counting of all labeled profiles that were present in each section was performed to determine the number of labeled profiles that contained NTR2, NT, or FG. The analyzed sections were separated by 200–300 µm to minimize the possibility of counting the same cell more than once.

### Electron microscopy analysis

In group 2, the rats recovered for 3 days after BDA injection into the PAG, followed by recovery for an additional 3 days after HRP injection into the cervical spinal cord. The rats were deeply anesthetized and then transcardially perfused with 0.9% saline, followed by 0.1 M PB containing 4% paraformaldehyde, 0.05% glutaraldehyde, and 0.2% picric acid.

Transverse sections from the midbrain to the cervical cord were generated using a vibratome (Microslicer DTM-1000, DSK, Kyoto, Japan) at a 50-µm thickness and were serially collected into four dishes. All sections in the four dishes were processed for the histochemical detection of HRP using the tetramethylbenzidine-sodium tungstate (TMB-ST) method (Gu et al., 1992). The HRP reaction products were intensified using a DAB/cobalt/H_2_O_2_ solution (Rye et al., 1984). The sections in the first dish were mounted on gelatin-coated slides and were used to observe the HRP injection sites in the spinal cord.

The reacted sections of the lower medulla oblongata from the second, third, and fourth dishes were freeze-thawed in liquid nitrogen to enhance the penetration of the antibodies. Details of the immuno-electron microscopy procedures were described in our previous reports (Li et al., 2002b). Briefly, the sections were incubated in 20% Tris-buffered saline (TBS)-NDS for 1 h to block non-specific immunoreactivity. The sections in the second, third, and fourth dishes were incubated for 24 h at 4°C in primary antibodies to NT/NTR2, NTR2, and NT (Table 1), respectively. Then, the second, third, and fourth dishes were incubated overnight in biotinylated donkey anti-rat IgG/goat anti-rabbit IgG conjugated to 1.4-nm gold particles for NTR2, goat anti-rabbit IgG conjugated to 1.4-nm gold particles for NTR2, or goat anti-rat IgG conjugated to 1.4-nm gold particles for NT (Table 1), respectively, at 48°C. Subsequently, all sections were processed according to the following steps: (1) post-fixation with glutaraldehyde, (2) silver enhancement using the HQ Silver Kit (Nanoprobes, Stony Brook, NY, USA), (3) incubation with the ABC kit (Vector), (4) reaction with DAB tetrahydrochloride and H_2_O_2_, (5) osmification; and (6) counterstaining with uranyl acetate. Ultrathin sections at a 70-nm thickness were prepared from the superficial laminae of the SDH, mounted on single-slot grids, and examined under an electron microscope (JEM1440, Tokyo, Japan).

## Results

### Light microscopy analysis

#### Distribution of the BDA-labeled fibers and the FG-labeled neurons in the RVM

**Figure 1:**
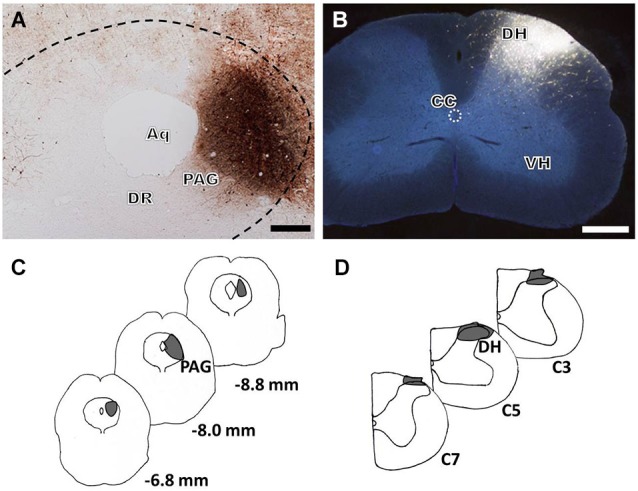
**Bright-field and fluorescence photomicrographs showing the BDA injection site in the LPAG (A, scale bar = 100 μm) and the FG injection site in the cervical SDH (B, scale bar = 200 μm)**. Camera lucida drawings show the rostro-caudal extent of the BDA **(C)** and FG **(D)** injection sites at different levels, indicated by the blackened areas on the drawings. The numbers **(C)** correspond to the distance in millimeters (mm) posterior to bregma in the brain, and C3, C5, and C7 **(D)** indicate the corresponding segments of the cervical spinal cord. Aq: aqueduct; DR: dorsal raphe nucleus; DH: dorsal horn; VH: ventral horn; CC: central canal.

For immunofluorescence labeling analysis of groups 1 and 3, the anterograde tracer BDA was injected into the PAG (Figures 1A,C) and the retrograde tracer FG was injected into the cervical SDH (Figures 1B,D). The BDA-labeled fibers and axon terminals were widely scattered from the rostral to the caudal ventral portion of the brainstem, including a dense distribution in the RVM, especially within the nucleus raphe magnus (NRM). Injections of FG into the right cervical SDH resulted in many retrogradely labeled spinally projecting neurons in the RVM (results not shown).

#### PAG efferents projecting to the RVM contact spinally projecting NTR2-IR neurons

**Figure 2:**
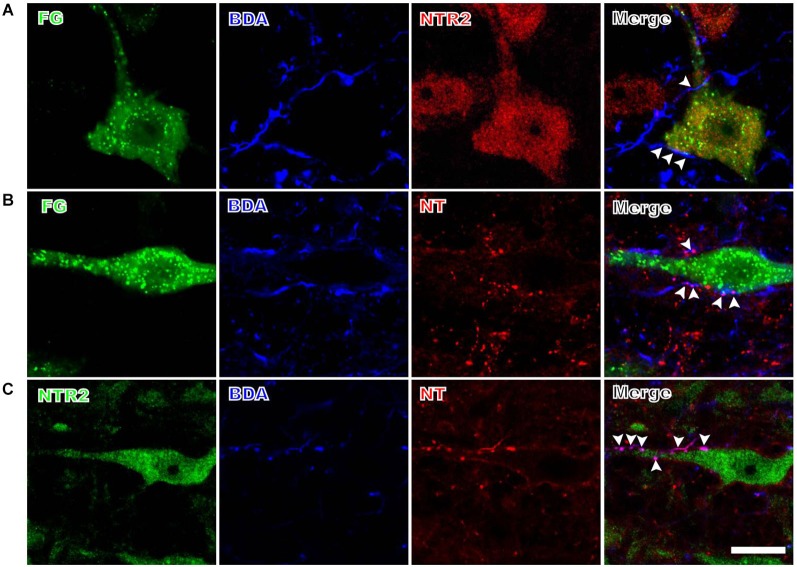
**Representative fluorescence photomicrographs displaying the close contacts between anterogradely BDA-labeled fibers and terminals and FG/NTR2 double-labeled neurons (A), between BDA-labeled NTergic fibers and terminals and retrogradely FG-labeled neurons (B), and between BDA-labeled NTergic fibers and terminals and NTR2-IR neurons (C)**. The white arrowheads indicate the locations of the BDA-labeled and/or NT-IR terminals and fibers in close contact with FG-labeled, NT/FG co-labeled, or NTR2-IR neuronal somata and dendritic processes. Scale bar = 20 µm.

The anterogradely labeled fibers and terminals originating from the PAG contacted the retrogradely FG-labeled spinally-projecting neurons in the RVM (Figure 2). Although BDA was injected into a relatively limited portion of the PAG, the BDA-labeled fibers and terminals were found in close apposition to approximately 32% (80/248) of the FG-labeled spinally projecting neurons that were analyzed. Moreover, 59% (146/248) of the retrogradely labeled spinally projecting neurons displayed NTR2-IR staining (Figure 2A). The BDA-labeled fibers and terminals in the RVM often targeted these NTR2-IR neurons (Figure 2A). Specifically, 51 of the 80 spinally projecting neurons (63%) that were apposed to PAG fibers also displayed NTR2 immunoreactivity.

#### NTergic fibers and terminals from the PAG target NT-IR spinally projecting neurons in the RVM

Many of the PAG efferent fibers projecting to the RVM displayed NT immunoreactivity (Figure 2B). These NTergic fibers and terminals from the PAG frequently targeted FG-labeled neurons in the RVM. In the current study, approximately half (51%) of the fibers and terminals from the PAG contacted FG-labeled spinally projecting neurons that also displayed NT immunoreactivity. In contrast, of the 80 PAG efferent fibers that targeted FG-labeled cells, 39 (49%) did not display NT immunoreactivity.

#### NTergic fibers and terminals from the PAG contact NTR2-IR neurons in the RVM

The BDA-labeled fibers and terminals were often found to contact NTR2-IR neurons in the RVM (Figure 2C). A total of 93 BDA-labeled axon terminals were found in close apposition to 253 NTR2-IR neurons; 43% (40/93) of these BDA-labeled axon terminals displayed NT immunoreactivity.

#### Spinally projecting NTR2-IR neurons display NT immunoreactivity in the RVM

**Figure 3:**
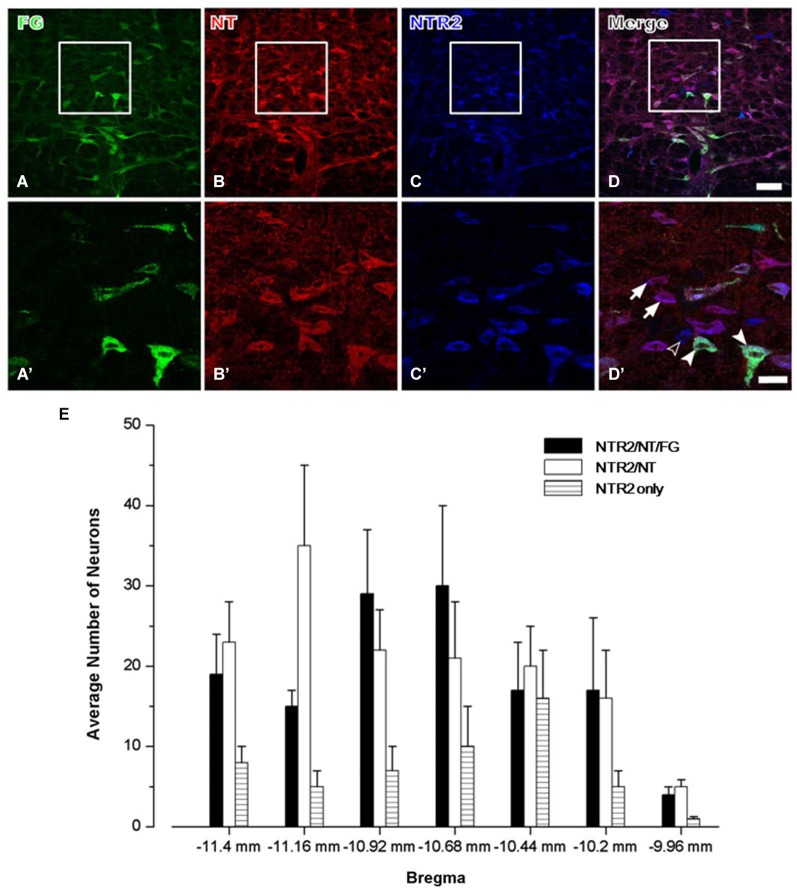
**Representative fluorescence photomicrographs showing FG- (A, green), NT- (B, red), and NTR2- (C, blue) triple-labeled neurons in the RVM**. The images from **(A–C)** are merged in **(D)**. The rectangular areas in **(A–D)** were enlarged and displayed in **(A’–D’)**. The arrows indicate NTR2/NT double-labeled neurons; the unfilled arrowheads indicate NTR2-IR neurons; and the filled arrowheads indicate FG/NT/NTR2 triple-labeled neurons in the RVM **(D’)**. Scale bar = 80 µm (**A–D)** or 30 µm **(A’–D’)**. **(E)** The mean number of NTR2-IR or NTR2/NT double-labeled or NTR2/NT/FG triple-labeled neurons in the RVM at seven rostro-caudal levels (from 11.4 mm to 9.96 mm caudal to bregma) revealed via immunofluorescence histochemical triple-staining for NTR2, NT, and FG (*n* = 5 rats).

In group 3, triple-labeling immunofluorescence histochemical staining was conducted to determine the exact neurochemical properties of the FG-labeled neurons on which NTR2 is expressed. It has been reported that NTR1 is expressed almost exclusively on serotonergic neurons in the RVM, approximately 50% of which project directly to the dorsal horn of the spinal cord (Buhler et al., 2005). Therefore, in this study, we primarily focused on the neurochemical properties of the NTR2-IR RVM neurons. After colchicine treatment, the neuropeptide NT is largely retained in the somata of the RVM neurons. Taking advantage of this method, we found that 80.65% of the NTR2-IR neurons in the RVM were also immunoreactive for NT-IR. Of these NT/NTR2 double-labeled neurons, approximately half (47.54%) were labeled with FG injected into the cervical dorsal horn (Figure 3).

#### NTR2-IR RVM neurons express both NT and 5-HT

**Figure 4:**
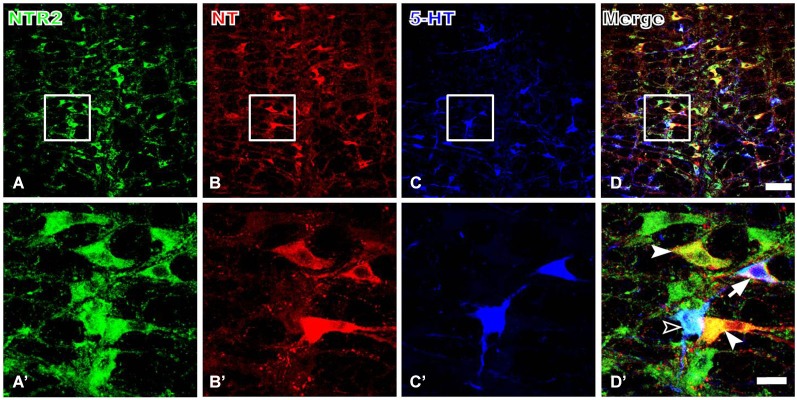
**Representative fluorescence photomicrographs showing NTR2- (A, green), NT- (B, red), and 5-HT- (C, blue) triple-labeled neurons in the RVM**. The images from **(A–C)** are merged in **(D)**. The rectangular areas in **(A–D)** were enlarged and displayed in **(A’–D’)**. The filled arrowheads indicate NTR2/NT double-labeled neurons; the unfilled arrowheads indicate NTR2/5-HT double-labeled neurons; and the arrows indicate NTR2/NT/5-HT tripe-labeled neurons in the RVM **(D’)**. Scale bar = 80 µm **(A–D)** or 25 µm **(A’–D’)**.

Because serotonergic neurons were previously demonstrated to be involved in the descending pain control pathway, especially those localized in the RVM, we further investigated the relationship between NT and 5-HT expression and whether the NTR2-IR RVM neurons also express 5-HT. Our triple-labeling immunofluorescence histochemical staining results suggested that the NTR2-IR RVM neurons express both NT and 5-HT. Specifically, 5-HT immunoreactivity was strongly detected in the neuronal somata and their processes (Figure 4). Approximately 80.65% and 45.42% of the NTR2-IR neurons were immunopositive for NT and 5-HT, respectively. NTR2/NT/5-HT triple-labeled neurons accounted for 33.43% of all NTR2-IR RVM neurons (Table 2).

**Table 2:** The number of neurons immunopositive for NTR2, NT, 5-HT, and all three markers in the RVM*.

Markers
(1) NTR2^+^ neurons (mean ± S.E.M)	139 ± 5.39
(2) NT^+^ neurons (mean ± S.E.M)	129 ± 4.98
(3) 5-HT^+^ neurons (mean ± S.E.M)	149 ± 3.37
(4) NTR2/NT^+^ neurons (mean ± S.E.M)	114 ± 5.28
(5) NTR2/5-HT^+^ neurons (mean ± S.E.M)	101 ± 3.91
(6) NTR2/NT/5-HT^+^ neurons (mean ± S.E.M)	45 ± 2.15
(7) (4)/(1) × 100%	80.65%
(8) (5)/(1) × 100%	45.42%
(9) (6)/(1) × 100%	33.43%

*The counts in rats were performed on six sections from a series of every fourth 30-μm-thick section.

### Electron microscopy analysis

To generate potent morphological evidence for the connections observed via light microscopy analysis, electron microscopy analysis was performed to detect synaptic connections and to elucidate the neurochemical properties of the synapses on such an NT-NTR2 participated pathway.

#### NT-IR terminals form synapses with spinally projecting NTR2-IR RVM neurons

**Figure 5:**
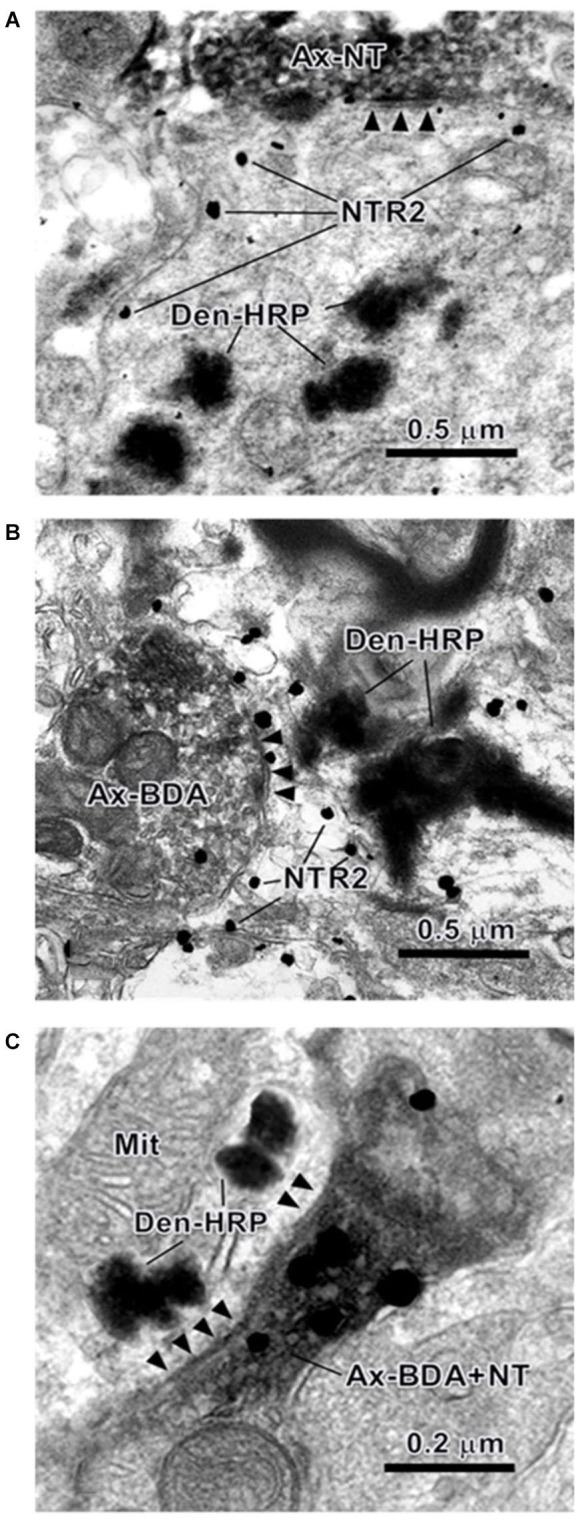
**Electron photomicrographs showing the synaptic connections in the RVM revealed using the triple-labeling method**. An NT-IR axon terminal (Ax-NT) **(A)** or a BDA-labeled axon terminal (Ax-BDA) **(B)** synapses on a dendrite that is retrogradely labeled with HRP (Den-HRP) and immunopositively stained for NTR2. The synaptic contact between an NT/BDA double-labeled axon terminal (Ax-BDA+NT) and an HRP-labeled dendritic profile (Den-HRP) is also shown **(C)**. The arrowheads indicate the post-synaptic membranes. Mit: mitochondria.

Triple-staining for NT-IR, NTR2-IR, and HRP was performed to detect the synaptic connections and neurochemical properties of the NT-IR synaptic buttons, the NTR2-IR neurons, and the spinally projecting RVM neurons (Figure 5A). Under electron microscopy, the HRP-labeled spinally projecting neurons were detected based on the presence of highly electron-dense clumps of crystalline material and occasionally the presence of amorphous puncta in the cytoplasm and in large dendrites. NTR2 immunoreactivity was determined based on the presence of the immunogold-silver grains, which were distributed in the cytoplasm, dendrites, and axonal fibers and terminals of the NTR2-IR neurons. The dendrites or somata containing more than four immunogold-silver grain particles in their postsynaptic structure were considered to indicate NTR2-IR neurons. The NT-IR axonal terminals, typically filled with synaptic vesicles, were characterized based on the presence of electron-dense DAB reaction products that adhered to the outer surface of organelles, such as mitochondria, synaptic vesicles, and the inner surface of the plasma membrane. It was found that NT-IR axonal terminals containing DAB reaction products formed asymmetric synaptic connections with the dendritic profiles or neuronal somata containing HRP-labeling products and/or immunogold-silver-enhanced particles corresponding to NTR2 (Figure 5A).

#### The BDA-labeled terminals from the PAG formed synapses with spinally projecting NTR2-IR RVM neurons

Another aim of this EM study was to confirm the synaptic connections between the BDA-labeled descending axonal terminals and the spinally projecting NTR2-IR RVM neurons using a triple-labeling method (Figure 5B). At the ultrastructural level, BDA-labeled products, HRP-labeled structures, and NTR2-IR gold particles enhanced using silver solution displayed the same morphological appearance as described above. Under electron microscopy, it was observed that the BDA-labeled axonal terminals filled with DAB reaction products formed asymmetric synapses with dendritic profiles or somata containing both HRP-labeled products and NTR2-IR gold particles. These results indicate that the axon terminals from the PAG formed synaptic contacts with spinally projecting NTR2-IR RVM neurons.

#### NT-IR terminals from the PAG formed synapses with spinally projecting RVM neurons

Moreover, after BDA was injected into the PAG and HRP was injected into the SDH, under electron microscopy, synaptic connections were detected between NT-IR terminals originating from PAG containing both anterogradely BDA-labeled DAB reaction products and NT-IR gold particles and retrogradely HRP-labeled neuronal somata and dendritic processes in the RVM (Figure 5C). These results indicate that the BDA/NT double-labeled terminals originating from the PAG form synapses with HRP-labeled dendrites or somata that send projecting fibers to the spinal cord.

## Discussion

In the current study, we demonstrated that NTR2-IR RVM neurons that project to the SDH receive NTergic descending projecting fibers and terminals originating from the PAG based on morphological analysis via tract tracing, immunofluorescence and immunohistochemical staining, and light and electron microscopy. A subset of the neurons projecting from both the PAG and RVM appear to utilize NT as a neurotransmitter. Furthermore, approximately one-third of the NTR2-IR neurons in the RVM that sent projection fibers to the spinal cord expressed both NT and 5-HT. These findings suggest that NT-NTR2-mediated signaling is involved in an NTergic PAG-RVM-SDH descending pathway.

Due to the importance of the PAG-RVM-SDH descending pain control pathway in mammals, many studies have been performed to elucidate the mechanisms underlying this critical pathway. Consistent with previous reports (Morgan et al., 2008), we confirmed that terminals from the PAG synapsed on spinally projecting RVM neurons. There are many NT-IR neurons in the PAG, and a major NTergic projection from the PAG to the RVM contributes to the descending modulation of nociception (Beitz et al., 1983). In addition, NT-IR neuronal somata and terminals are detected in the RVM (Dobner, 2006). Previous studies have also shown that NTR2 mRNA and protein are expressed in the RVM (Maeno et al., 2004). Our present data integrate all these independent findings and show that NTR2-IR neurons in the RVM receive NT-IR projections originating from the PAG and, in turn, send projections to the superficial laminae (laminae I and II) of the SDH. The present results provide morphological evidence for an NTergic PAG-RVM-SDH descending pathway. These morphological data are consistent with those of previous neuropharmacology studies, suggesting that NTR2 in the RVM is essential for NT-induced analgesia. Microinjection of the selective NTR2 agonist beta-lactotensin (β-LT) or the non-selective NTR2 agonist levocabastine into the RVM produces antinociception based on tail-flick tests (Smith et al., 1997; Buhler et al., 2008). Similarly, NT-induced antinociception is not completely blocked by the selective NTR1 antagonist SR48692 (Dubuc et al., 1994; Buhler et al., 2005).

Our present data also show that RVM neurons displaying both NTR2- and NT-immunopositive labeling send fibers projecting to the SDH. Specifically, more than 80% of the NTR2-IR neurons in the RVM were found to express NT, approximately half of which project to the SDH. These NTergic projections from the RVM to the SDH may partially account for the NT-IR fibers and terminals in the spinal cord, which were detected previously (Seybold and Elde, 1982). The other NT/NTR2-IR neurons that are not labeled with FG may either contact local circuit neurons within the RVM, thus regulating NT-mediated signal transduction, or may represent SDH-projecting neurons that fail to absorb the tracer. It has been reported that the antinociceptive response induced by the NTR2 agonist β-LT is reduced via intrathecal administration of yohimbine, an α_2_-adrenoceptor antagonist (Buhler et al., 2008), indicating that the effects of NTR2 are likely partially mediated by noradrenalin release. However, to our knowledge, there are no noradrenergic neurons present in the RVM. Tract tracing combined with immunohistochemical identification of noradrenergic A7 neurons that directly project to the spinal cord demonstrated that the majority of the neurons in the RVM, including the NRM and the nucleus reticularis gigantocellularis pars α, project to the A7 cell group and the surrounding area of the dorsolateral pontine tegmentum (Clark and Proudfit, 1991; Holden and Proudfit, 1998). Therefore, we propose that the remaining NTR2-IR neurons observed in our study directly or indirectly project to the noradrenergic A7 cell group, thereby modulating noradrenalin release in the spinal cord.

It is well known that 5-HT serves as a primary neurotransmitter for the descending projection from the RVM to the spinal cord. The present results showed that 5-HT is expressed in NTR2-IR neurons. Approximately half (45.42%) of the NTR2-IR RVM neurons displayed 5-HT immunoreactivity, and approximately one-third (33.43%) of the NTR2/NT-IR neurons expressed 5-HT. These results indicated that NT and 5-HT are co-expressed in NTR2-IR neurons in the RVM and, thus, are most likely co-released simultaneously in the SDH to modulate nociceptive transmission. Our previous study revealed that 5-HT exerts an analgesic effect by potentiating glycine release in the superficial laminae neurons via the protein kinase C pathway (Li et al., 2002a). The detection of serotonergic neurons that were not labeled with NTR2 or NT may be due to the following three reasons. First, they may express NTR1 and account for another portion of serotonergic neurons in the RVM that descend to the SDH. Second, the procedures for the tissue treatment or the processes of triple-labeling immunohistochemistry may also affect the detectability of the antibodies. Third, not all of the SDH-projecting serotonergic neurons are modulated by the neurotensinergic system.

Previous studies have indicated that both NTR1 and NTR2 are required for NT-induced antinociception. The results of previous studies using NTR1 knockout mice have demonstrated an important role for NTR1 in NT-induced antinociception based on the hot plate test (Pettibone et al., 2002) but not the acetic acid-induced writhing test (Remaury et al., 2002). Pharmacological antagonist and agonist studies provide evidence that NTR2 is also involved in the analgesic effect of NT. The analgesic effects produced by intracerebroventricular administration of NT are not attenuated by the NTR1 antagonist SR 48692 but are blocked by SR 142948A, which antagonizes both NTR1 and NTR2 (Dubuc et al., 1994; Gully et al., 1997). Selectively activating NTR2 via injection of β-LT into the RVM of rats produces an antinociceptive response based on the tail withdrawal test (Buhler et al., 2008). In addition, an antisense inhibition experiment further supported the evidence for the involvement of NTR2 in NT-induced antinociception. Central administration of phosphorothioate antisense oligonucleotides against NTR2 clearly inhibited the NT-mediated antinociceptive response based on the acetic acid-induced writhing test (Dubuc et al., 1999). Taken together, it appears that both NTR1 and NTR2 are required for different aspects of NT-induced analgesia, as NTR1 is specific for hot plate tests and NTR2 is specific for writhing tests. However, recently, two different lines of evidence have challenged this sharp distinction between these two receptors. NT79 and levocabsatine (both NTR2-selective agonists) reduced writhing in NTR1^−/−^ mice, whereas NT72 (an NTR1-selective agonist) exerted a significant analgesic effect on NTR2^−/−^ mice, indicating that both NTR1 and NTR2 are involved in NT-mediated analgesia in a visceral pain model using acetic acid-induced writhing tests (Smith et al., 2012). Moreover, it has been found that both NTR1 and NTR2 suppress the persistent inflammatory pain responses observed after intraplantar injection of formalin into rats (Roussy et al., 2008, 2009).

**Figure 6:**
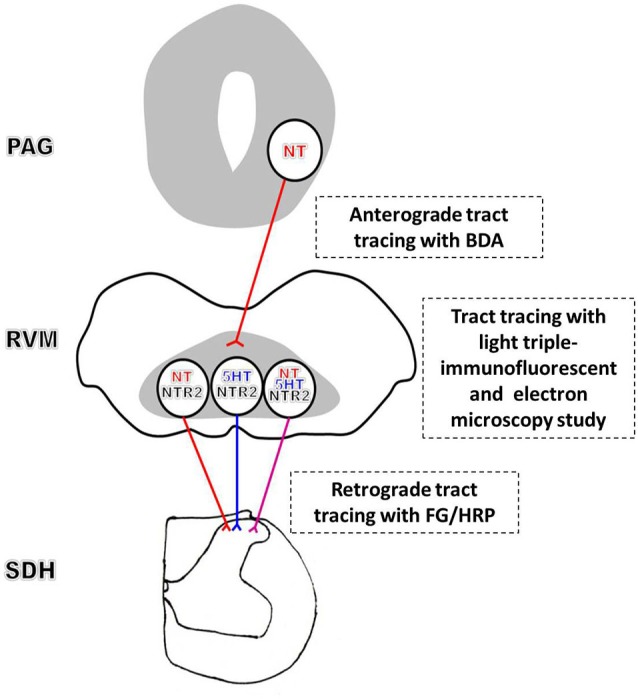
**Schematic diagram of the proposed NT-NTR2 system in the PAG-RVM-SDH pathway**. The efferent NT-expressing axon terminals from the PAG synapse on spinally projecting RVM neurons. The NT-expressing nerve fibers from the PAG may primarily synapse on NTergic neurons in the RVM that display NTR2-immunopositive staining, which, in turn, project to the SDH. In addition, some of the NTR2-IR neurons, but relatively fewer than the NT-IR neurons, express 5-HT. Approximately one-third of these NTR2-IR neurons appear to utilize both NT and 5-HT as their neurotransmitters. Thus, NT and 5-HT may be co-released in the SDH to modulate nociceptive transmission.

Combining our current results with the previous findings, we conclude that an NTergic PAG-RVM-SDH descending pathway significantly contributes to NT-induced analgesia. Specifically, convergent stimulation activates NTergic neurons in the PAG, in turn exciting the spinally projecting NT/NTR2 co-expressing RVM neurons, which most likely simultaneously increases NT and 5-HT release in the spinal cord, thereby modulating pain transmission (Figure 6). In contrast to the NTergic PAG-RVM descending pathway, the involvement of NTR2 in the NTergic pathway from the PAG to the SDH most likely indicates that NTR2 is critical for NT-mediated analgesia. Furthermore, the current study may bridge the analgesic effects of NT between the spinal and supraspinal levels. Nonetheless, the present study also has some limitations. Due to the limitations of the immunohistochemical staining method, quadruple or quintuple staining is not available to simultaneously investigate all of the relationships and connections described in the present study. Moreover, to fully establish the functional role of and the downstream molecular mechanisms underlying NT and NTR2 activity in the NT-NTR2 pathway, experiments combining morphology, physiology, and molecular biology must be performed to determine whether and how these labeled neurons and terminals function in different circuits at various levels.

## Conclusion

There is an indirect NTergic PAG-RVM-SDH descending pathway from the PAG to the SDH. Both NT and NTR2 are critical for this pathway. This pathway may represent an important component of the descending system involved in the modulation of nociceptive transmission.

## Conflict of interest statement

The authors declare that the research was conducted in the absence of any commercial or financial relationships that could be construed as a potential conflict of interest.

## References

[B1] Basbaum A. I. Fields H. L. (1984). Endogenous pain control systems: brainstem spinal pathways and endorphin circuitry. Annu. Rev. Neurosci. 7, 309–338 10.1146/annurev.neuro.7.1.309 6143527

[B2] Beitz A. J. Mullett M. A. Weiner L. L. (1983). The periaqueductal gray projections to the rat spinal trigeminal, raphe magnus, gigantocellular pars alpha and paragigantocellular nuclei arise from separate neurons. Brain Res. 288, 307–314 10.1016/0006-8993(83)90108-7 6198027

[B3] Buhler A. V. Choi J. Proudfit H. K. Gebhart G. F. (2005). Neurotensin activation of the NTR1 on spinally-projecting serotonergic neurons in the rostral ventromedial medulla is antinociceptive. Pain 114, 285–294 10.1016/j.pain.2004.12.031 15733655

[B4] Buhler A. V. Proudfit H. K. Gebhart G. F. (2008). Neurotensin-produced antinociception in the rostral ventromedial medulla is partially mediated by spinal cord norepinephrine. Pain 135, 280–290 10.1016/j.pain.2007.06.010 17664042 PMC2423280

[B5] Carraway R. Leeman S. E. (1973). The isolation of a new hypotensive peptide, neurotensin, from bovine hypothalami. J. Biol. Chem. 248, 6854–6861 4745447

[B6] Clark F. M. Proudfit H. K. (1991). Projections of neurons in the ventromedial medulla to pontine catecholamine cell groups involved in the modulation of nociception. Brain Res. 540, 105–115 10.1016/0006-8993(91)90496-i 1711394

[B7] Dobner P. R. (2006). Neurotensin and pain modulation. Peptides 27, 2405–2414 10.1016/j.peptides.2006.04.025 16870306

[B8] Dong Y. Li J. Zhang F. Li Y. (2011). Nociceptive afferents to the premotor neurons that send axons simultaneously to the facial and hypoglossal motoneurons by means of axon collaterals. PLoS One 6:e25615 10.1371/journal.pone.0025615 21980505 PMC3183065

[B9] Dubuc I. Costentin J. Terranova J. P. Barnouin M. C. Soubrié P. Le Fur G. (1994). The nonpeptide neurotensin antagonist, SR 48692, used as a tool to reveal putative neurotensin receptor subtypes. Br. J. Pharmacol. 112, 352–354 10.1111/j.1476-5381.1994.tb13077.x 8075852 PMC1910364

[B10] Dubuc I. Sarret P. Labbé-Jullié C. Botto J. M. Honoré E. Bourdel E. (1999). Identification of the receptor subtype involved in the analgesic effect of neurotensin. J. Neurosci. 19, 503–510 9870978 10.1523/JNEUROSCI.19-01-00503.1999 PMC6782393

[B11] Gu Y. Chen Y. Ye L. (1992). Electron microscopical demonstration of horseradish peroxidase by use of tetramethylbenzidine as chromogen and sodium tungstate as stabilizer (TMB-ST method): a tracing method with high sensitivity and well preserved ultrastructural tissue. J. Neurosci. Methods 42, 1–10 10.1016/0165-0270(92)90129-2 1405726

[B12] Gully D. Canton M. Boigegrain R. Jeanjean F. Molimard J. C. Poncelet M. (1993). Biochemical and pharmacological profile of a potent and selective nonpeptide antagonist of the neurotensin receptor. Proc. Natl. Acad. Sci. U S A 90, 65–69 10.1073/pnas.90.1.65 8380498 PMC45600

[B13] Gully D. Labeeuw B. Boigegrain R. Oury-Donat F. Bachy A. Poncelet M. (1997). Biochemical and pharmacological activities of SR 142948A, a new potent neurotensin receptor antagonist. J. Pharmacol. Exp. Ther. 280, 802–812 9023294

[B14] Holden J. E. Proudfit H. K. (1998). Enkephalin neurons that project to the A7 catecholamine cell group are located in nuclei that modulate nociception: ventromedial medulla. Neuroscience 83, 929–947 10.1016/s0306-4522(97)00437-5 9483575

[B15] Li H. Kang J. F. Li Y. Q. (2002a). Serotonin potentiation of glycine-activated whole-cell currents in the superficial laminae neurons of the rat spinal dorsal horn is mediated by protein kinase C. Brain Res. Bull. 58, 593–600 10.1016/s0361-9230(02)00826-2 12372564

[B16] Li Y. Q. Tao F. S. Okamoto K. Nomura S. Kaneko T. Mizuno N. (2002b). The supratrigeminal region of the rat sends GABA/glycine-cocontaining axon terminals to the motor trigeminal nucleus on the contralateral side. Neurosci. Lett. 330, 13–16 10.1016/s0304-3940(02)00711-5 12213623

[B17] Lü B. C. Li H. Chen T. Huo F. Q. Zhang T. Li Y. Q. (2009). Endomorphin 1- and endomorphin 2-containing neurons in nucleus tractus solitarii send axons to the parabrachial nuclei in the rat. Anat. Rec. (Hoboken) 292, 488–497 10.1002/ar.20847 19301276

[B18] Maeno H. Yamada K. Santo-Yamada Y. Aoki K. Sun Y. J. Sato E. (2004). Comparison of mice deficient in the high- or low-affinity neurotensin receptors, Ntsr1 or Ntsr2, reveals a novel function for Ntsr2 in thermal nociception. Brain Res. 998, 122–129 10.1016/j.brainres.2003.11.039 14725975

[B19] Mazella J. (2001). Sortilin/neurotensin receptor-3: a new tool to investigate neurotensin signaling and cellular trafficking? Cell. Signal. 13, 1–6 10.1016/s0898-6568(00)00130-3 11257441

[B20] Mazella J. Vincent J. P. (2006). Functional roles of the NTS2 and NTS3 receptors. Peptides 27, 2469–2475 10.1016/j.peptides.2006.04.026 16872720

[B21] Mitchell V. A. Kawahara H. Vaughan C. W. (2009). Neurotensin inhibition of GABAergic transmission via mGluR-induced endocannabinoid signalling in rat periaqueductal grey. J. Physiol. 587, 2511–2520 10.1113/jphysiol.2008.167429 19359367 PMC2714017

[B22] Mogil J. S. (2013). Pain genetics: past, present and future. Trends Genet. 28, 258–266 10.1016/j.tig.2012.02.004 22464640

[B23] Morgan M. M. Whittier K. L. Hegarty D. M. Aicher S. A. (2008). Periaqueductal gray neurons project to spinally projecting GABAergic neurons in the rostral ventromedial medulla. Pain 140, 376–386 10.1016/j.pain.2008.09.009 18926635 PMC2704017

[B24] Niu L. Chen T. Wang Y. Y. Li Y. Q. (2009). Neurochemical phenotypes of endomorphin-2-containing neurons in vagal nodose neurons of the adult rat. Neurochem. Int. 55, 542–551 10.1016/j.neuint.2009.05.010 19463881

[B25] Paxinos G. Watson C. (2005). The Rat Brain in Stereotaxic Coordinates. New York: Academic Press

[B26] Pettibone D. J. Hess J. F. Hey P. J. Jacobson M. A. Leviten M. Lis E. V. (2002). The effects of deleting the mouse neurotensin receptor NTR1 on central and peripheral responses to neurotensin. J. Pharmacol. Exp. Ther. 300, 305–313 10.1124/jpet.300.1.305 11752130

[B27] Remaury A. Vita N. Gendreau S. Jung M. Arnone M. Poncelet M. (2002). Targeted inactivation of the neurotensin type 1 receptor reveals its role in body temperature control and feeding behavior but not in analgesia. Brain Res. 953, 63–72 10.1016/s0006-8993(02)03271-7 12384239

[B28] Roussy G. Dansereau M. A. Baudisson S. Ezzoubaa F. Belleville K. Beaudet N. (2009). Evidence for a role of NTS2 receptors in the modulation of tonic pain sensitivity. Mol. Pain 5:38 10.1186/1744-8069-5-38 19580660 PMC2714839

[B29] Roussy G. Dansereau M. A. Doré-Savard L. Belleville K. Beaudet N. Richelson E. (2008). Spinal NTS1 receptors regulate nociceptive signaling in a rat formalin tonic pain model. J. Neurochem. 105, 1100–1114 10.1111/j.1471-4159.2007.05205.x 18182046

[B30] Rye D. B. Saper C. B. Wainer B. H. (1984). Stabilization of the tetramethylbenzidine (TMB) reaction product: application for retrograde and anterograde tracing and combination with immunohistochemistry. J. Histochem. Cytochem. 32, 1145–1153 10.1177/32.11.6548485 6548485

[B31] Seybold V. S. Elde R. P. (1982). Neurotensin immunoreactivity in the superficial laminae of the dorsal horn of the rat: I. Light microscopic studies of cell bodies and proximal dendrites. J. Comp. Neurol. 205, 89–100 10.1002/cne.902050109 7040502

[B32] Smith K. E. Boules M. Williams K. Richelson E. (2012). NTS1 and NTS2 mediate analgesia following neurotensin analog treatment in a mouse model for visceral pain. Behav. Brain Res. 232, 93–97 10.1016/j.bbr.2012.03.044 22504145

[B33] Smith D. J. Hawranko A. A. Monroe P. J. Gully D. Urban M. O. Craig C. R. (1997). Dose-dependent pain-facilitatory and -inhibitory actions of neurotensin are revealed by SR 48692, a nonpeptide neurotensin antagonist: influence on the antinociceptive effect of morphine. J. Pharmacol. Exp. Ther. 282, 899–908 9262357

[B34] Taylor B. K. Basbaum A. I. (2003). Systemic morphine-induced release of serotonin in the rostroventral medulla is not mimicked by morphine microinjection into the periaqueductal gray. J. Neurochem. 86, 1129–1141 10.1046/j.1471-4159.2003.01907.x 12911621

[B35] Tsukahara S. Yamanouchi K. (2003). Distribution of glutamic acid decarboxylase, neurotensin, enkephalin, neuropeptide Y and cholecystokinin neurons in the septo-preoptic region of male rats. J. Reprod. Dev. 49, 67–77 10.1262/jrd.49.67 14967951

[B36] Vincent J. P. Mazella J. Kitabgi P. (1999). Neurotensin and neurotensin receptors. Trends Pharmacol. Sci. 20, 302–309 10.1016/S0165-6147(99)01357-7 10390649

[B37] White J. F. Noinaj N. Shibata Y. Love J. Kloss B. Xu F. (2012). Structure of the agonist-bound neurotensin receptor. Nature 490, 508–513 10.1038/nature11558 23051748 PMC3482300

[B38] Yang K. Ma W. L. Feng Y. P. Dong Y. X. Li Y. Q. (2002). Origins of GABA(B) receptor-like immunoreactive terminals in the rat spinal dorsal horn. Brain Res. Bull. 58, 499–507 10.1016/s0361-9230(02)00824-9 12242103

